# Syringaresinol protects against hypoxia/reoxygenation-induced cardiomyocytes injury and death by destabilization of HIF-1α in a FOXO3-dependent mechanism

**DOI:** 10.18632/oncotarget.2723

**Published:** 2014-11-06

**Authors:** Siyoung Cho, Miook Cho, Juewon Kim, Matt Kaeberlein, Sang Jun Lee, Yousin Suh

**Affiliations:** ^1^ R&D Unit, Amorepacific Corporation, Yongin-si, Gyeonggi-do, Korea; ^2^ Department of Genetics, Albert Einstein College of Medicine, Bronx, NY, USA; ^3^ Department of Pathology, University of Washington, Seattle, WA, USA; ^4^ Department of Medicine, Diabetes Research and Training Center, Albert Einstein College of Medicine, Bronx, NY, USA; ^5^ Institute for Aging Research, Diabetes Research and Training Center, Albert Einstein College of Medicine, Bronx, NY, USA

**Keywords:** HIF-1α, Syringaresinol, FOXO3, ischemia/reperfusion, hypoxia/reoxygenation, cardiomyocytes

## Abstract

Hypoxia-inducible factor 1 (HIF-1) is a master regulator of hypoxic response and has been a prime therapeutic target for ischemia/reperfusion (I/R)-derived myocardial dysfunction and tissue damage. There is also increasing evidence that HIF-1 plays a central role in regulating aging, both through interactions with key longevity factors including Sirtuins and mTOR, as well as by directly promoting longevity in *Caenorhabditis elegans*. We investigated a novel function and the underlying mechanism of syringaresinol, a lignan compound, in modulation of HIF-1 and protection against cellular damage and death in a cardiomyocyte model of I/R injury. Syringaresinol caused destabilization of HIF-1α following H/R and then protected against hypoxia/reoxygenation (H/R)-induced cellular damage, apoptosis, and mitochondrial dysfunction in a dose-dependent manner. Knock-down of *FOXO3* by specific siRNAs completely abolished the ability of syringaresinol to inhibit HIF-1 stabilization and apoptosis caused by H/R. Syringaresinol stimulated the nuclear localization and activity of FOXO3 leading to increased expression of antioxidant genes and decreased levels of reactive oxygen species (ROS) following H/R. Our results provide a new mechanistic insight into a functional role of syringaresinol against H/R-induced cardiomyocyte injury and death. The degradation of HIF-1α through activation of FOXO3 is a potential therapeutic strategy for ischemia-related diseases.

## INTRODUCTION

Proper adaptation to endogenous and exogenous stressors is critical for cells and organisms to survive [1]. Among the stresses that cells unavoidably encounter, oxygen availability is emerging as an important factor influencing health and lifespan in invertebrate and mammalian systems [2, 3]. Short-term exposure to reduced oxygen availability can lead to a beneficial metabolic adaptation associated with increased cellular and organismal survival [4-6], while prolonged exposure of cells to hypoxia leads to DNA damage, cell death, and contributes to many diseases including diabetes, atherosclerosis and cardiovascular diseases (CVD) [7]. In particular, ischemia in which blood supply to tissue is restricted causes a shortage of oxygen and impaired cellular metabolism triggering ischemic heart diseases, a leading cause of mortality worldwide [8]. In multicellular eukaryotes, the primary system for adapting to low oxygen levels is the hypoxic response pathway [9-11]. A key player in this pathway is the hypoxia induction factor 1 (HIF-1), a master regulator of the response to hypoxia that regulates the expression of a broad range of genes that facilitate the adaptation to, and survival of cells to low oxygen environments [12, 13]. Under normoxia, HIF-1α is hydroxylated, targeted by von Hippel-Lindau protein, ubiquitinated, and finally degraded by 26S proteasomes [14]. In hypoxic conditions, the hydroxylation modification declines and HIF-1α is stabilized for its transcriptional activities [15].

**Figure 1:**
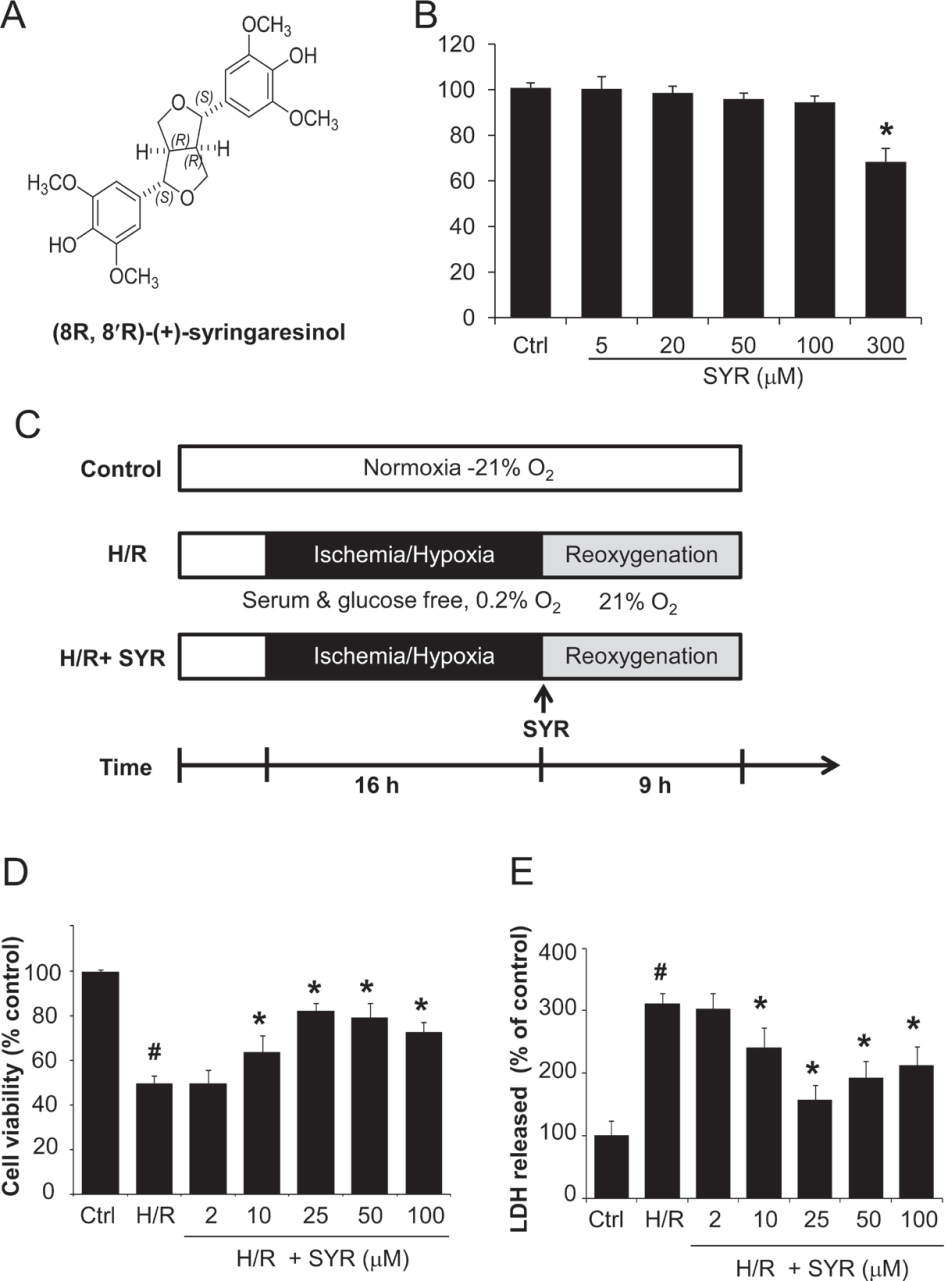
Syringaresinol promotes cell survival and reduces cell damage after H/R in myocardial H9c2 cells (A) Chemical structure of syringaresinol. (B) Cytotoxicity of syringaresinol was tested by treating H9c2 cells with the indicated concentration of syringaresinol for 24 h. (C) Experimental design. To mimic the ischemic injury *in vitro*, H9c2 cells were incubated in a medium without glucose and serum for 16 h of hypoxia followed by reoxygenation in a medium with 10% FBS for 9 h. Syringaresinol was added to the medium at the beginning of the reoxygenation phase. Ctrl:control; H/R: hypoxia/reoxygenation; SYR: syringaresinol. (D) H9c2 cells were exposed to hypoxia for 16 h and treated with different concentrations of syringaresinol at the beginning of reoxygenation. After 9 h of reoxygenation, cell viability was determined by MTT assay. (E) The release of LDH in culture medium was determined at the end of reoxygenation. Results were expressed as percentages of control and presented as mean ± SD for six independent experiments. #P < 0.001 versus control group, *P < 0.001 versus H/R treatment group.

Several studies have indicated that HIF-1 activity is modulated by both SIRT1 and FOXO3. SIRT1 represses HIF-1α activity by deacetylation of HIF-1α [16, 17]. FOXO3 inhibits HIF-1α activity and HIF-1α-induced apoptosis by directly binding to HIF-1α [18], or via induction of a negative regulator of HIF-1α, CBP/P300-interacting transactivator with Glu/Asp-rich carboxy-terminal domain 2 (CITED2) [19, 20]. Recently, our studies have shown that syringaresinol (4,4′-(1S,3aR,4S,6aR)-tetrahydro-1H,3H-furo[3,4-c]furan-1,4-diylbis(2,6-dimethoxyphenol) (its chemical structure is shown in Figure 1A), isolated from *panax ginseng* berry pulp, activates *SIRT1* gene expression leading to delayed cellular senescence and improved endothelial cell function in endothelial cells [21]. The beneficia1 effects exerted by syringaresinol were dependent on FOXO3, which we have shown to bind the SIRT1 promoter in a sequence-specific manner and activate its expression in response to syringaresinol treatment. In addition, syringaresinol has been shown to inhibit inflammation in lipopolysaccharide treated macrophages and oxidative injury of endothelial cells [22-24].

In the present study, we investigated the role of syringaresinol in modulation of HIF-1 destabilization and I/R injury using the well-established tissue culture model of I/R injury that causes cardiomyocyte death. We found that syringaresinol promoted rapid degradation of HIF-1α during a H/R treatment in a FOXO3-dependent mechanism, and attenuated H/R-induced cardiomyocyte death. These data indicate that upon reoxygenation, rapid degradation of HIF-1α via FOXO3 activation is important for cell survival after I/R injury.

## RESULTS

### Syringaresinol Alleviates Hypoxia/Reoxygenation injury

We first examined the cytotoxicity of syringaresinol in the cardiomyocyte cell line, H9c2. The cells were treated with different concentrations (5, 20, 50, 100 and 300 μM) of syringaresinol for 24 h, at which time cell viability was assessed. Increasing doses of syringaresinol up to 100 μM did not cause cellular cytotoxicity in H9c2 cells as shown by MTT assay (Figure 1B).

We then used a well-established model of I/R injury, a H/R treatment that causes cardiomyocyte death in the H9c2 cell line [20]. H9c2 cells underwent 16 h of hypoxia followed by 9 h of reoxygenation (Figure 1C). Exposure of H9c2 cells to H/R led to a significant decrease in cell viability, while syringaresinol treatment increased survival of cardiomyocyte cells undergoing H/R challenge in a dose-dependent manner (Figure 1D). The treatment of 25 μM syringaresinol resulted in maximal protective effects against H/R injury. Since lactate dehydrogenase (LDH) leakage is widely used as a marker of cellular damage, cardiomyocyte cells injury was assessed by determining LDH activity in culture medium at the end of reoxygenation. LDH leakage increased in the H/R group compared with the control group, but was significantly decreased by syringaresinol treatment (Figure 1E).

### Syringaresinol Inhibits Apoptosis Induced by H/R in Myocardial H9c2 Cells

**Figure 2:**
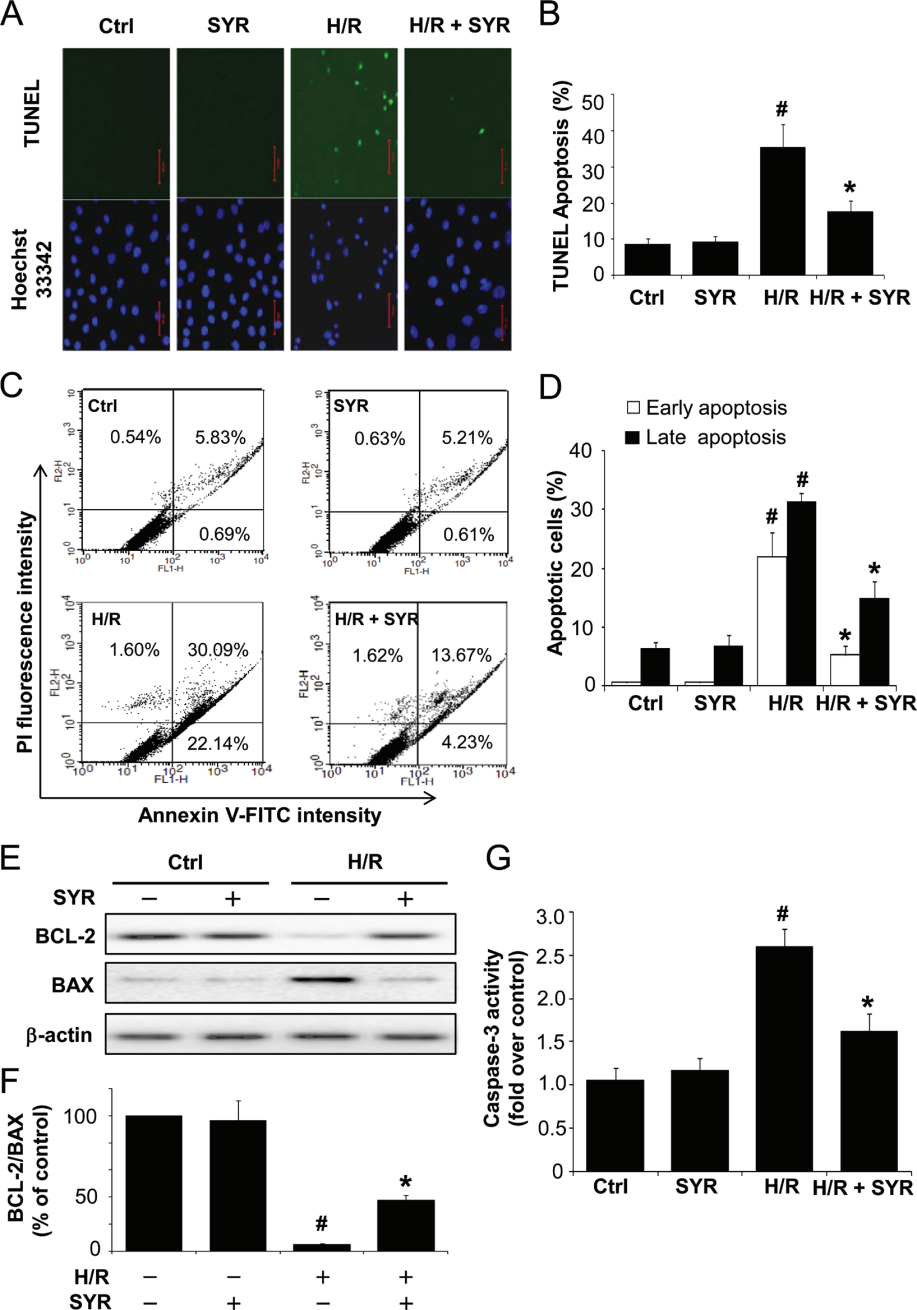
Syringaresinol protects cardiomyocytes against H/R-induced apoptosis H9c2 cells were exposed to hypoxia stress for 16 h and treated with 25 μM syringaresinol during reoxygenation. After 9 h of reoxygenation, cell death was determined by TUNEL staining and flow-cytometric analysis (A) Representative images of TUNEL-positive cells (green) and Hoechst 33342 counterstaining (blue). Scale bar: 50 μm. (B) The relative proportion of TUNEL-positive cells. (C) Flow cytometry analysis of the annexin V-FITC/PI staining. (D) Quantification histograms indicate the percentages of early (annexin V-FITC positive and PI negative) and late (annexin V-FITC positive and PI positive) apoptotic cells. (E) Expression levels of apoptotic-related proteins, BCL-2 and BAX were examined by Western blot. Actin levels are shown as loading controls. (F) BCL-2/BAX ratios were assessed. (G) Caspase-3 activity was detected using a commercial kit as described in Materials and Methods. Results are presented as fold-changes over the control group. All results are representative or means ± SD of six independent experiments. Ctrl: control; H/R: hypoxia/reoxygenation; SYR: syringaresinol. #P < 0.001 versus control group, *P < 0.001 versus H/R treatment group.

We next measured the effects of syringaresinol on H/R-induced apoptosis by terminal deoxyuncleotidyl transferase-mediated dUTP nick end-labeling (TUNEL) assay and flow cytometric analysis. After H/R injury, the apoptosis index of the H/R group was markedly increased compared to the control group, whereas the apoptosis index was significantly decreased by 25 μM syringaresinol treatment compared to the non-treated H/R group (Figure 2A-D). To further confirm the protective effects of syringaresinol against H/R-induced apoptosis, the expression levels of apoptosis-related proteins such as BCL-2 and BAX were examined using Western blot analysis. The BCL-2/BAX ratio decreased in the H9c2 cells exposed to H/R and this decrease was greatly attenuated by 25 μM syringaresinol treatment (Figure 2E and F). In addition, while caspase 3 activity, a key stimulator of cell apoptosis in intrinsic pathway, was significantly increased (by 1.7 fold) after H/R (Figure 2G), the H/R-induced caspase 3 activation was inhibited by syringaresinol.

### Syringaresinol Suppresses HIF-1α Stabilization and Protects Myocardial H9c2 Cells against Mitochondrial Dysfunction Following H/R

**Figure 3:**
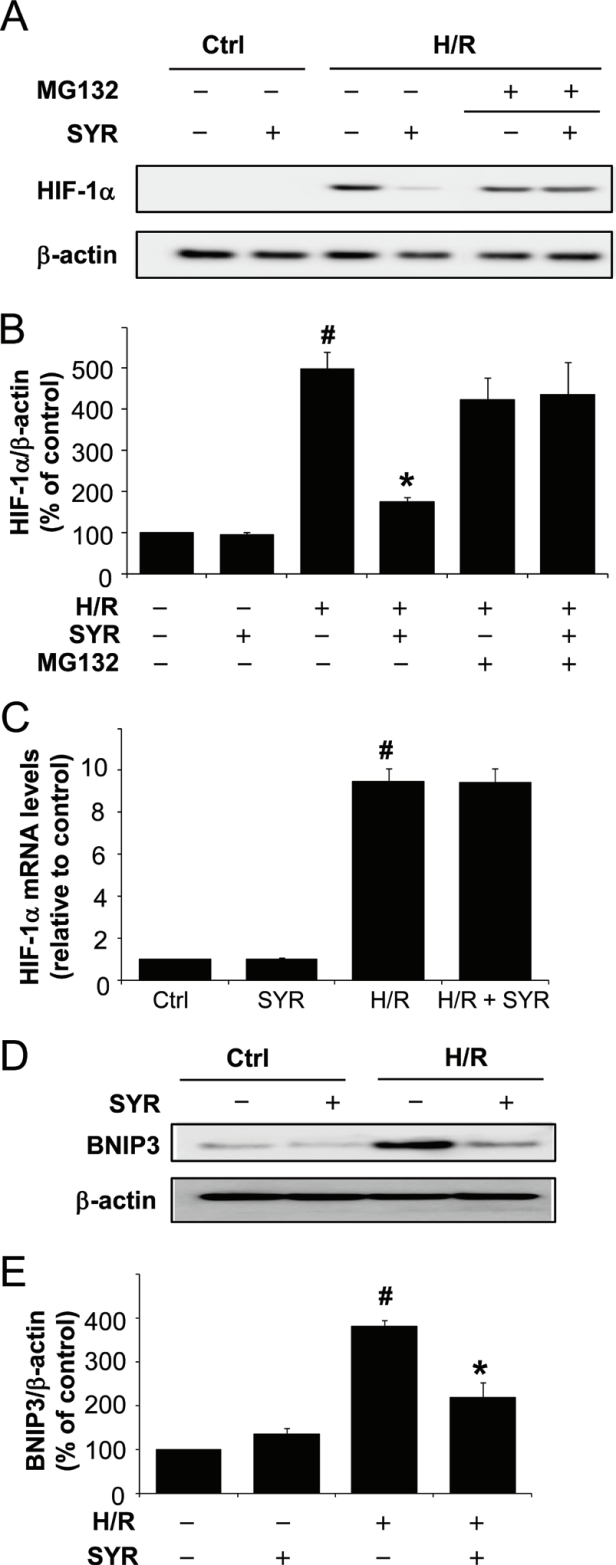
Syringaresinol promotes the degradation of HIF-1 and blocks the upregulation of a HIF-1α target gene, Bnip3 during H/R H9c2 cells were exposed to hypoxia stress for 16 h followed by treatment with 25 μM syringaresinol and reoxygenation for a further 9 h. 25 μM MG132 was added for the final 4 h. (A) Expression of HIF-1α protein was determined by Western blot. (B) Quantification of HIF-1α protein levels over actin as loading control. (C) HIF-1α mRNA levels were determined by real-time RT-PCR. The levels were quantified and normalized using GAPDH. (D) Expression of BNIP3 protein was determined by Western blot. (E) Quantification of BNIP3 protein levels over actin as loading control. All results are representative or means ± SD of six independent experiments. #P < 0.001 versus control group, *P < 0.001 versus H/R treatment group. Ctrl:control; H/R: hypoxia/reoxygenation; SYR: syringaresinol.

We next investigated the molecular mechanism underlying the protective effects of syringaresinol against H/R-induced cardiomyocyte cells injury. We focused on effects of syringaresinol on HIF-1α because it is a master regulator of cellular responses to hypoxia. We found that the level of HIF-1α protein was markedly increased after H9c2 cells were exposed to H/R compared with that in the control group (Figure 3A and B), whereas syringaresinol treatment abolished HIF-1α induction by H/R. However, syringaresinol led to neither a substantial reduction in HIF-1α mRNA (Figure 3C) nor blockage of H/R-induced increase in HIF-1α protein levels in the presence of MG132, an inhibitor of 26S proteasome complex (Figure 3A and B), suggesting that syringaresinol regulates the stability of HIF-1α protein. Consistently, while expression of a HIF-1α target, BNIP3, was markedly increased in H9c2 cells after H/R compared with that after normoxia, syringaresinol treatment suppressed the H/R-induced increase in BNIP3 protein expression (Figure 3D and E). BNIP3 expression levels in absence of H/R condition did not exhibit a statistically significant difference in syringaresinol-treated cells as compared to untreated cells (p=0.09).

**Figure 4:**
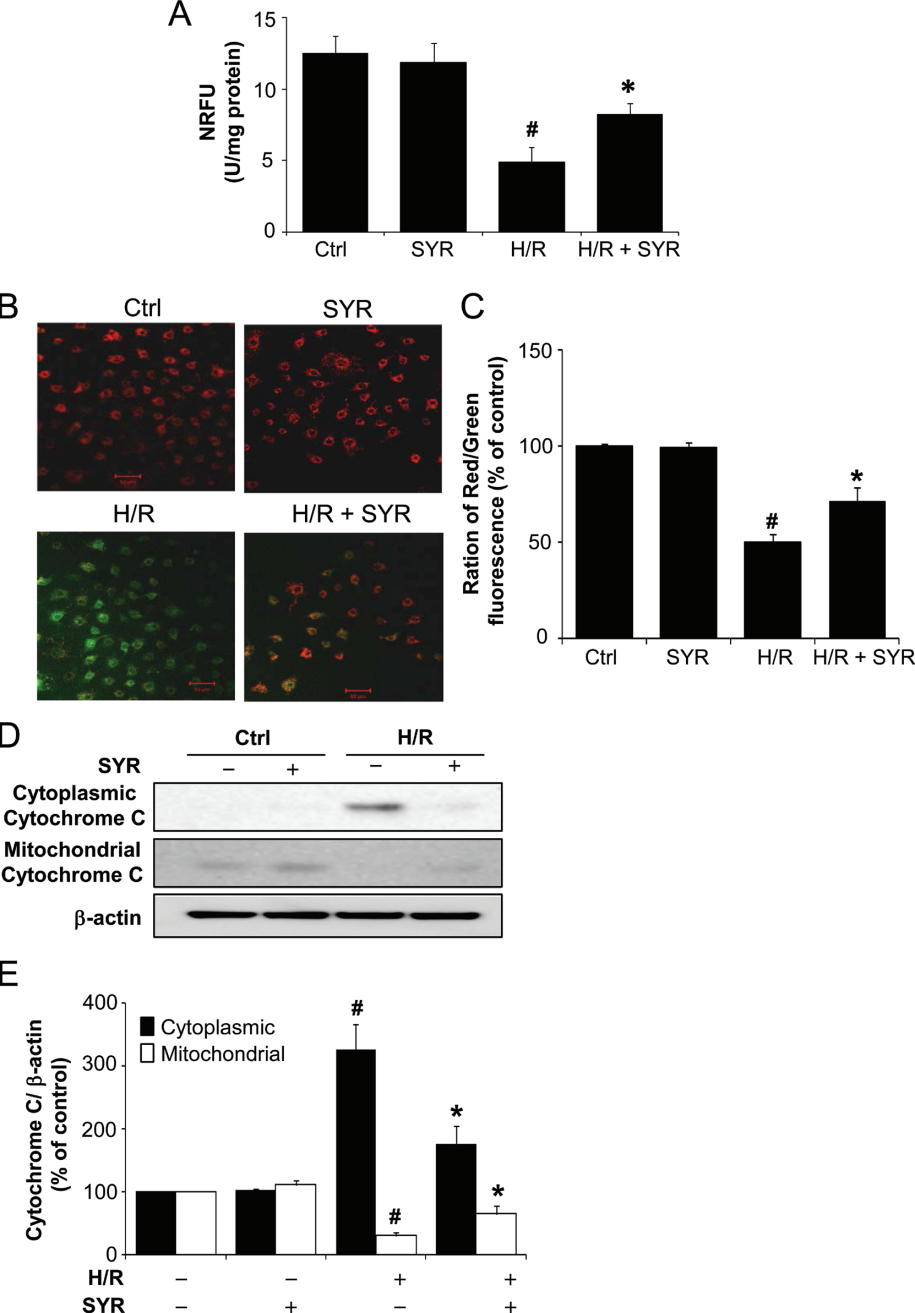
Syringaresinol prevents mitochondrial dysfunction induced by H/R (A) The influence of syringaresinol on the opening of mPTP in H9c2 exposed to H/R was assayed using the calcein-cobalt quenching method. The NRFU of calcein measurements were compared among different treatment groups of H9c2. (B) The effects of syringaresinol on H/R-induced dissipation of mitochondrial membrane potential (ΔΨm) were determined by JC-1 staining. Red fluorescence is from JC-1 aggregates in healthy mitochondria with polarized inner membranes, while green fluorescence is emitted by cytosolic JC-1 monomers and indicates ΔΨm dissipation. Scale bar: 50 μm. (C) A bar graph showing the ratio of red to green intensity indicates the changes in ΔΨm. (D) Levels of cytochrom c in the mitochondrial and cytosolic fraction were determined using Western blot analysis. (E) Quantification of cytochrome c levels over actin. All results are representative or means ± SD of six independent experiments. #P < 0.001 versus control group, *P < 0.001 versus H/R treatment group. NRFU: normalized relative fluorescence units. Ctrl:control; H/R: hypoxia/reoxygenation; SYR: syringaresinol.

BNIIP3 has been shown to induce the activation of BAX/BAK, opening of the mitochondrial permeability transition pore (mPTP), increased production of reactive oxygen species (ROS), and cell death [26]. To test the effects of syringaresinol on mPTP opening after H/R, we monitored the distribution of green fluorescence emitted from calcein as readout of the intact mPTP using the calcein-cobalt method. In agreement with the BNIP3 expression levels, a significant decrease in mitochondrial fluorescence was observed after H/R but treatment with syringaresinol counteracted the decrease leading to higher normalized relative fluorescence units (NRFU) of calcein than the non-treated group (Figure 4A), suggesting that syringaresinol inhibited H/R induced mPTP opening, possibly by regulating expression of several genes involved in mPTP formation. We further determined changes in mitochondrial membrane potential (ΔΨm) using the reporter dye JC-1. JC-1 exhibits potential-dependent accumulation in mitochondria of which depolarization is indicated by the decreased ratio of red/green intensity. Exposure of H9c2 cells to H/R resulted in dissipation of ΔΨm (Figure 4B). Cells treated with syringaresinol demonstrated attenuation in the dissipation of ΔΨm caused by H/R (Figure 4C). Moreover, Western blot revealed that H/R led to an accumulation of cytochrome c in the cytosol (Figure 4D and 4E), while it was significantly reduced when the cells were treated with syringaresinol during reoxygenation. Taken together, these results indicate that syringaresinol allows the proteasome to more rapidly degrade HIF-1α upon reoxygenation and exerts protective effects against H/R-induced mitochondrial dysfunction.

### FOXO3 Is Required for Destabilization of HIF-1α and Protection of Myocardial H9c2 Cells exposed to H/R by Syringaresinol

**Figure 5:**
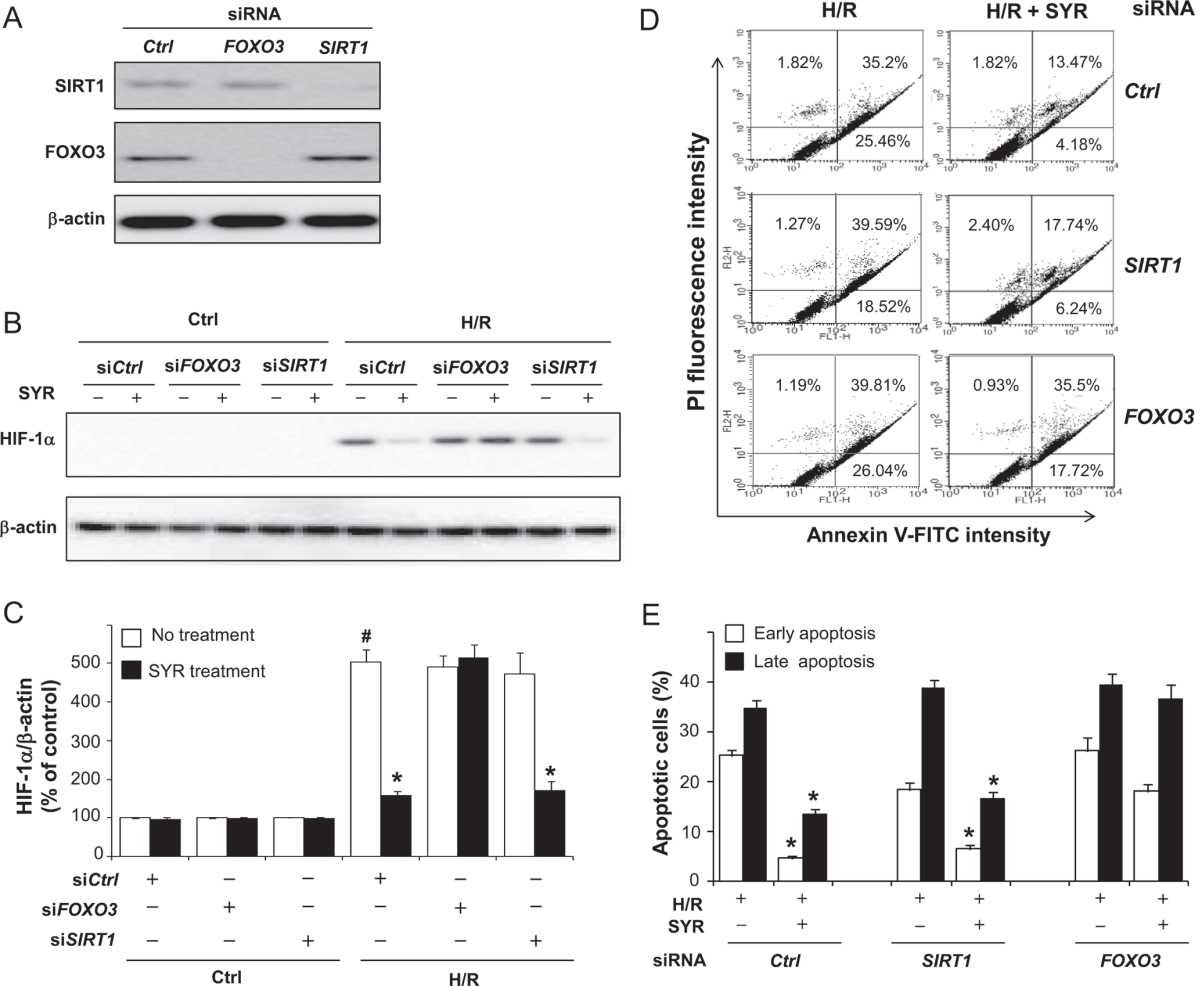
Syringaresinol-induced destabilization of HIF-1α and protection against cell death is mediated by FOXO3 H9c2 cells were transfected with control, *SIRT1* or *FOXO3* siRNA. After 24hr, cells were exposed to H/R in the presence and absence of syringaresinol. (A) At 48 h after transfection of siRNAs, suppression of SIRT1 and FOXO3 expression was validated by Western blot analysis. (B) The expression levels of HIF-1α protein. (C) Quantification of HIF-1α protein levels over actin. (D) Cell death in the cultures was determined by flow cytometry. (E) Quantification of the percentages of early (annexin V-FITC positive and PI negative) and late (annexin V-FITC positive and PI positive) apoptotic cells. All results are representative or means ± SD of six independent experiments. #P < 0.001 versus control group, *P < 0.001 versus H/R treatment group. Ctrl:control; H/R: hypoxia/reoxygenation; SYR: syringaresinol.

**Figure 6:**
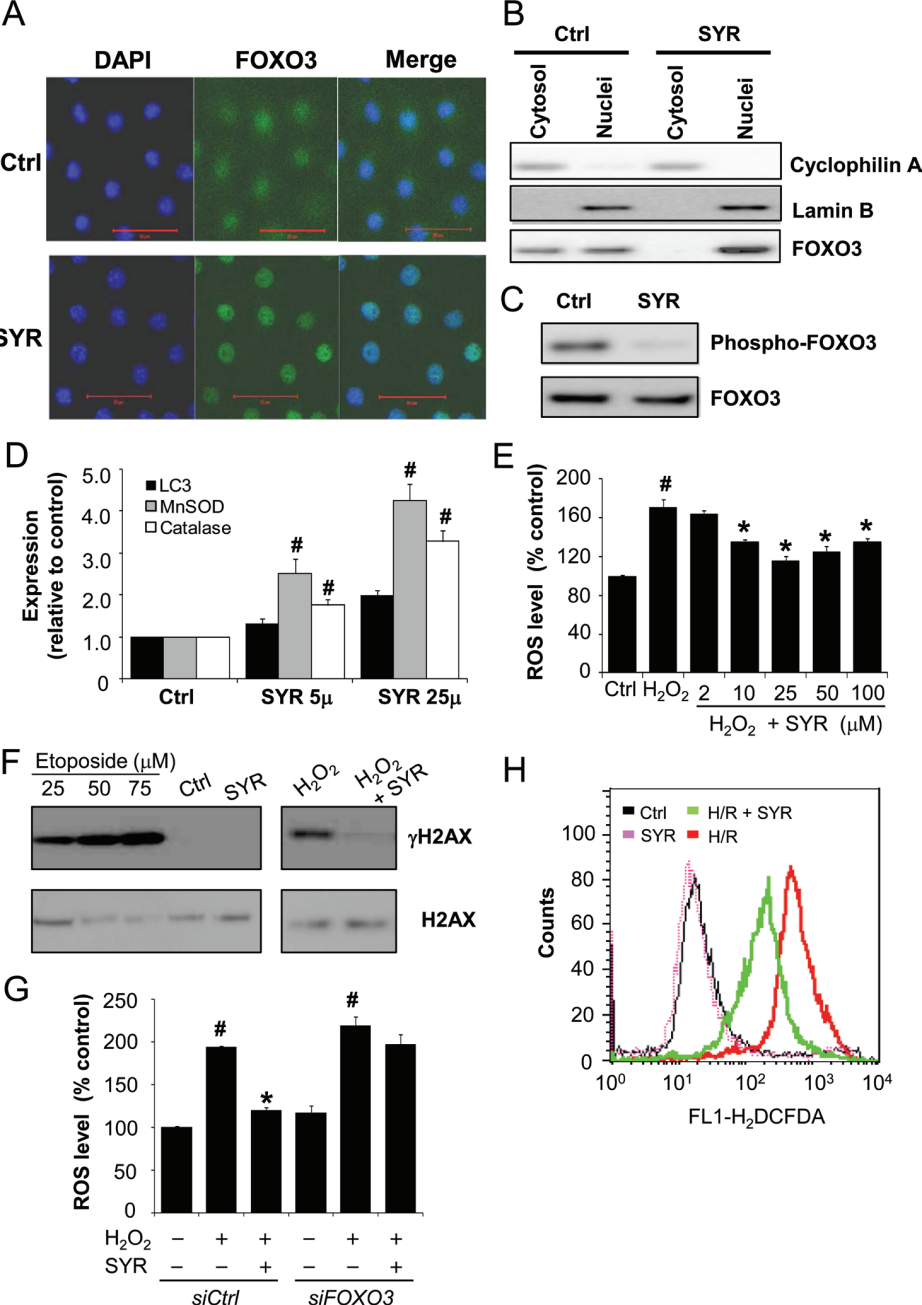
Syringaresinol induces FOXO3 nuclear accumulation, upregulation of FOXO3 target genes expression, and decreased production of ROS H9c2 cells were treated with syringaresionl for 24 h. (A) Cellular localization of FOXO3 were determined by immunofluorescence. Cells were fixed and stained with anti-FOXO3 (green) and DNA was stained by 4′,6-diamidino-2-phenylindole (DAPI) (blue). Bar: 50μm. (B) Equal cell equivalents of nucleus and cytosol were separated by gel electrophoresis and processed for Western blotting with the indicated antibodies. (C) Levels of phosphorylation of FOXO3 were examined by Western blotting. (D) The expression of the indicated FOXO3 target genes were measured by real time PCR. The levels were quantified and normalized by GAPDH. (E) H9c2 cells were pre-treated with increasing concentrations of syringaresinol (0, 2, 10, 25, 50 and 1000 μM) for 9 h and then exposed to 150 μM H_2_O_2_ for 6 h. Cellular ROS levels (percentage compared to Ctrl) were analyzed by carboxy-H_2_DCFDA staining followed by flow cytometry. (F) H9c2 cells were treated with 25 μM Syringaresinol for 15 min or 9 h prior to incubation with or without 150 μM H_2_O_2_ for 6 h. γH2AX formation (the phosphorylated form of H2AX) was examined by Western blotting with anti-γH2AX antibody. For positive control, cells were treated with indicated concentration of etoposide. (G) H9c2 cells were transfected with control or *FOXO3* siRNA. After 24 h cells were pre-treated with or without 25 μM syringaresinol for 9 h and then exposed to 150 μM H_2_O_2_ for 6 h. Cellular ROS levels (percentage compared to Ctrl) were analyzed by carboxy-H_2_DCFDA staining followed by flow cytometry. (H) ROS levels in H2c9 exposed to normoxia, SYR, H/R or H/R+SYR were determined by DCFDA staining followed by flow cytometry. All results are representative or means ± SD of six independent experiments. #P < 0.001 versus control group *P < 0.001 versus H_2_O_2_ treatment group. Ctrl:control; H/R: hypoxia/reoxygenation; SYR: syringaresinol.

Given that syringaresinol activates *SIRT1* gene expression through FOXO3 (21), and that HIF-1α is negatively regulated by both SIRT1 and FOXO3 [16-20], we tested whether the ability of syringaresinol to promote HIF-1α degradation after H/R depends on either SIRT1 or FOXO3. H9c2 cells were transfected with a control, *SIRT1* or *FOXO3* siRNA (Figure 5A), cultured under H/R in the presence or absence of syringaresinol, and evaluated for cell survival and the levels of HIF-1α accumulation. Knockdown of *SIRT1* had no influence on the syringaresinol-induced acceleration of HIF-1α destabilization and its anti-apoptotic effects (Figure 5B and C). In contrast, knockdown of *FOXO3* completely abolished the ability of syringaresinol to inhibit HIF-1α stabilization and apoptosis caused by H/R (Figure 5B and C). Consistently, FOXO3 knockdown abolished the syringaresinol-induced change in BCL-2/BAX ratio after H/R (Supplementary Figure 1). To test whether FOXO3 activity is regulated by syringaresinol, we examined subcellular localization and phosphorylation of FoxO3 and the expression of FOXO target genes. Syringaresinol treatment resulted in predominant nuclear localization of FOXO3 indicative of activation, compared with uniform distribution of FOXO3 throughout the H9c2 cells in non-treated conditions (Figure 6A and B). Consistently, syringaresinol treatment inhibited the phosphorylation of FOXO3 proteins (Figure 6C). In addition, mRNA levels of FOXO target genes, including MnSOD, catalase, and LC3 were significantly increased in syringaresinol-treated cardiomyocyte cells (Figure 6D).

Consistent with the induction of the antioxidant gene expression, treatment of syringaresinol led to decrease in H_2_O_2_-induced ROS levels in a dose-dependent manner (Figure 6E), with a dose of 25 μM syringaresinol leading to maximal effects as in protection against H/R injury (Figure 1C). Protective effects of syringaresinol against H_2_O_2_-induced oxidative damage were also confirmed by γH2AX (denoted the phosphorylated form of histone H2AX) assay (Figure 6F). Knock-down of *FOXO3* by siRNA abolished the effect of syringaresinol on H_2_O_2_-induced ROS levels (Figure 6G), suggesting that the protective effect was mediated through a FOXO3-dependent manner. Similarly, syringaresinol treatment led to reduction in ROS levels after H/R (Figure 6H). Taken together, our results indicate that FOXO3 plays an important role in mediating the protective effects of syringaresinol for cardiomyocytes cell line subjected to H/R through modulation of HIF-1α stability and its target gene expression.

## DISCUSSION

HIF-1 is a master regulator of the cellular response to low oxygen conditions such as myocardial I/R injury and stroke. In this study, we have shown that H/R-induced apoptosis was closely associated with delayed degradation of HIF-1α upon reoxygenation. Furthermore, syringaresinol protected myocardial H9c2 cells against H/R by promoting degradation of HIF-1α through FOXO3 activation.

While many HIF-dependent genes are involved in adaptation and survival of cells in low-oxygen environments, HIF activation has recently been reported to enhance cell death via induction of proapoptotic targets or via stabilization of p53 [27-30]. Especially, HIF-induced apoptosis is most common under very low oxygen concentrations (< 0.2 %) or prolonged hypoxia conditions [28]. Indeed, Wang et al demonstrated that HIF-1α triggered apoptosis following H/R through induction of Bnip3 and caspase-3 [31]. It has been shown that the ability of HIF-1α to promote apoptosis depends on multiple factors such as cell type, the level or duration of hypoxia, or the presence or absence of cofactors [19]. In our study, H9c2 cardiomyocyte cells were exposed to more severe and prolonged hypoxic conditions (incubation in serum and glucose-free medium at 0.2% O_2_ for 16 h). This condition led to pro-apoptotic HIF-1α accumulation and apoptosis induction, whereas stimulation of HIF-1α degradation by syringaresinol treatment rescued the apoptotic phenotype (Figure 3 and 4). Although it remains possible that syringaresinol also protects against H/R injury through additional HIF-1-independent mechanisms, our data are in agreement with previous studies [28, 32] supporting the model that HIF-1a stabilization and its delayed degradation upon reoxygenation plays an important role in triggering cell death in response to severe and prolonged hypoxic/reoxygenation conditions.

In agreement with previous studies [28, 32], our findings support the argument that HIF-1α stabilization and its delayed degradation upon reoxygenation plays an important role in triggering cell death in response to severe and prolonged hypoxic/reoxygenation conditions.

We also showed that the ability of syringaresinol in affecting HIF-1α destabilization and inhibiting apoptosis depends on FOXO3 in H/R-induced cardiomyocyte cell line. Several lines of evidence suggest that FOXO3 has critical functions in regulating cell survival in response to hypoxic stress or I/R injury. For example, mice with cardiomyocyte-specific FOXO1 and 3 deficiencies have reduced cardiac function, increased scar information, and increased apoptotic cells after acute I/R injury [20]. In *C. elegans*, the FOXO ortholog DAF-16 was required for survival under hypoxic conditions [33]. In the present study, we found that the activation of FOXO3 by syringaresinol resulted in a decrease in ROS production and a rapid increase in HIF-1α degradation, which was required for suppression of apoptosis induced by H/R (Figure 5 and 6). In the presence of oxygen, HIF-1α is degraded via the ubiquitin-proteasome pathway [15]. Recent studies indicate that activated FOXO3 increases the overall rate of protein degradation by coordinately activating both lysosomal and proteasomal pathways [34]. Therefore, syringaresinol may allow FOXO3 to coordinately activate both lysosomal and proteasomal proteolysis leading to accelerated breakdown of HIF-1α. Interestingly, knock-down of *SIRT1*, a pro-survival factor implicated in protection against HR-induced cell death [35] and expression of which is induced by syringaresinol in a FOXO3-dependent manner [21], did not affect syringaresinol-mediated suppression of HIF-1α stabilization and apoptosis induced by H/R, suggesting that syringaresinol protected cardiomyocyte cells from H/R injury independently of SIRT1.

In summary, we found that syringaresinol can provide protection of H9c2 cells against apoptosis induced by H/R, and that the anti-apoptotic effect depends, at least in part, on the acceleration of HIF-1α destabilization through FOXO3 activation. Our study demonstrates that induction of HIF-1α destabilization is beneficial and can be a therapeutic strategy against H/R injury. These results warrant further investigation of syringaresinol as a promising novel therapeutic agent against myocardial I/R injury and stroke.

## MATERIALS AND METHODS

### Chemicals

(+)-syringaresinol was synthesized at Hanchem (http://www.hanchem.net). The purity of the compound was > 99.7 %.

### Cell culture

The H9c2 cardiomyocyte cells were purchased from the American Tissue Type Collection and cultured in Dulbecco's modified Eagle's medium (DMEM, Invitrogen) supplemented with 10% fetal bovine serum (FBS), 100 units/ml penicillin and 100 μg/ml streptomycin in a humidified atmosphere of 5% CO_2_ at 37 °C. The cells were fed every 2-3 days, and sub-cultured once they reached 70-80% confluence.

### Stimulated ischemia/reperfusion model

The cells were seeded in a six-well plates at a density of 1 × 10^5^ cells per well. The cultures were grown at 37 °C for 24 h, and then the media were replaced with serum-free and glucose-free DMEM to prior to culture under hypoxia. Hypoxic stress was induced by incubation in an anaerobic Plexiglas chamber (Billups-Rothernberg, http://www.brincubator.com/hypoxiachamber.htm; 0.2% O_2_), saturated with 95% N_2_ and 5% CO_2_, at 37 °C. After 16 h of hypoxia, the cells were subjected to reoxygenation by changing the medium into 10% FBS-containing DMEM followed by incubation under normoxia (21% O_2_ and 5% CO_2_) for 9 h. This hypoxia/reoxygenation *in vitro* model resembles I/R *in vivo* [25]. For syringaresinol treatment, syringaresinol was dissolved in DMSO and added to the culture medium to the indicated final concentration at the beginning of the reoxygenation phase (Figure 1C).

### Measurement of cell viability

To determine cell viability, 3-[4,5-dimethylthiazol-2-yl]-2,5 diphenyltetrazolium bromide (MTT) assays were performed. After 24 h of treatment with different concentrations of syringaresinol, MTT solution was added into medium and incubated for an additional 4 h at 37 °C. The medium was removed and the formazan crystal, metabolized MTT, was dissolved with DMSO. The absorbance was measured at 490 nm using an Infinite^TM^ M200 Microplate Reader (Tecan). The reduction in optical density was considered to be the decrease in cell viability. Control group was considered 100% viable.

### Assay of LDH activity

To determine the amount of cell injury induced by H/R protocol, LDH activities in the culture media were measured using a Lactate Dehydrogenase Activity Assay Kit (Sigma-aldrich, cat #MAK066-1KT) according to the manufacturer's directions.

### TUNEL assay for apoptosis

Cell apoptosis was determined using TUNEL assay with an *in situ* cell death detection kit (Roche Applied Science, cat #11684795910) in accordance with the manufacturer's protocol. TUNEL-positive nuclei were counted in four non-overlapping fields per coverslip, and then were converted to percentage by comparing TUNEL-positive counts with the total cell nuclei determined by Hoechst33342 (Invitrogen, cat #H3570) counterstaining. Assay was performed in a blinded manner and the experiment was repeated for three times.

### Flow cytometric detection of apoptosis

Early apoptosis and necrosis were identified by double fluorescence staining using the Alexa Fluor® 488 annexin V/Dead Cell Apoptosis Kit according to the manufacturer's instructions (Invitrogen, cat #V1324). The H9c2 cardiomyoctes were harvested, washed twice with PBS, incubated with the 5 l FITC-Annexin V and 1 μl propidium iodide working solution (100 μg/ml) for 15 min in the dark at room temperature, and then cellular fluorescence was measured by flow cytometry analysis with a FACSCalibur Flow Cytometer (BD Biosciences).

### Measurement of caspase-3 activity

Caspase-3 activities were measured using the Fluorometric assay kit (BioVision, cat # K105-200) according to the maufacturer's instructions. The samples were read in a Flouoskan Ascent FL fluorometer (Thermo Fisher Scientific) using 400-nm excitation and 505-nm emission wavelengths, and the results were expressed as fold change over the control.

### Detection of Mitochondrial permeability transition pore (mPTP) opening

The opening mPTP of cardiomyocytes were detected by using the calcein-cobalt with a mPTP assay kit (Genmed Scientifics, cat #GMS10095) according to the manufacture's directions. Briefly, cardiomyocytes, seeded in 24-well plates, were washed with Reagent A, then incubated with Reagent B and C (1:50; 500 μl per well) at 37 C for 20 min, then washed twice with Reagent A again. Fluorescence intensity was measured using a InfiniteTM M200 Microplate Reader (Tecan). Cells were subsequently lysed in 20 μl of 0.1 M NaOH and protein concentration was measured using the Bradford Protein assay. The fluorescent signals were normalized to total protein content in the corresponding cell extract. Results were presented as normalized relative fluorescence units (NRFU; U/mg protein).

### Determination of mitochondrial membrane potential

5,5′,6,6′-tetrachloro-1,1′3,3′ tetraethylbenzimidazol yl-carbocyanine iodide (JC-1) (Invitrogen, cat #M34152) was used to determine the changes in mitochondrial membrane potential. After indicated treatment, the cells were loaded with JC-1 for 30 min at 37 °C, and images were obtained by using a Zeiss Axioplan-2 microscope and Northern Eclipse software (Empix). JC-1 fluorescence was analyzed by flow cytometry analysis with a FACS Calibur Flow Cytometer (BD Biosciences).

### Measurement of ROS generation

Cardiomyocytes were incubated in normoxia or subjected to H/R. Cells were equilibrated in 2μM carboxy-H_2_DCFDA (Invitrogen, cat #c400) in PBS/50mM HEPES for 30 minutes, followed by a recovery in DMEM + 10% FBS for 30 minutes, washed in PBS, trypsinized and resuspended in ice cold PBS with 10% FBS. Analysis was performed by flow cytometry on a FACS-Calibur (BD Biosciences). A minimum of 10,000 events were analyzed using FlowJo software. To investigate the effect of syringaresinol on the generation of intracellular ROS, the cells were pretreated with various concentrations of syringaresinol for 9 h prior to the addition of 150 μM H_2_O_2_ for 6 h. Then, the level of intracellular ROS was analyzed by carboxy-H_2_DCFDA staining followed by flow cytometry. DNA damage was analyzed using γH2AX assay with monoclonal antibody for H2AX histone (Upstate, cat #05-636).

### RNA extraction and Real-time PCR

Total RNA from cells was prepared with RNeasy mini kit from Qiagen (Qiagen, cat #74106). Reverse transcription was performed on 1 μg of total RNA using iScript cDNA Synthesis kit (Bio-Rad, cat #170-8891). RNA samples that were not reverse transcribed were used as “Non-RT control”. The pre-designed primers and probe sets of HIF-1, SOD2, catalase, Map1lc3b and glyceraldehyde-3-phosphate dehydrogenase were obtained from Applied Biosystems (assay identifications are as follows: Rn00577560_m1, Rn00690588_g1, Rn00560930_m1, Rn02132764_s1 and Rn01775763_g1) The reaction mixture was prepared using a Quantitect probe PCR kit (Qiagen, cat #204345) according to the manufacturer's instructions. Reaction and analysis were performed using the Rotor-Gene 3000 system (Corbett Research). All reactions were done in triplicate. The amount of mRNA was calculated by the comparative CT method.

### Subcellular fractionation

Cardiomyocytes were washed three times with ice-cold PBS and scraped into homogenization buffer (50 mM HEPES, pH 7.4, 255 mM sucrose, 1 mM EDTA) containing a protease inhibitor mixture (Sigma, cat #P8340). After homogenization with a glass homogenizer for 20 strokes, the homogenate was centrifuged at 1,000 x g for 10 min into supernatant 1 and pellet. The pellet was homogenized in 10 mM Tris buffer, pH 7.5, containing 300 mM sucrose, 1 mM EDTA, and protease inhibitor mixture and centrifuged 5,000 x g for 5 min. The resulting pellet was the nuclear fraction. Supernatant 1 was removed and centrifuged at 5,000 x g for 20 min to yield the pellet (mitochondria fraction) and supernatant 2 (cytosol fraction).

### Western blotting

Cells were lysed in RIPA buffer (PBS pH 7.4, containing 1% NP-40, 0.5% sodium deoxycholate, 0.1% SDS) with a protease inhibitor cocktail (Sigma-Aldrich). Forty micrograms of proteins were resolved on 4-12% NuPAGE gels run in an MES buffer system (Invitrogen, cat #NP0002) and transferred to PVDF membranes according to the manufacturer's protocol. Immunoreactive proteins were revealed by enhanced chemiluminescence with ECL Plus (Amersham, cat #RPN2133). Antibodies against BCL-2 (Cat #sc-7382), BAX (Cat #sc-7480), HIF1-α (Cat #sc-10790), BNIP3 (Cat #sc-1715), Cytochrome c (cyt c, cat #sc-13156), Lamin B (Cat #sc-374015) and SIRT1 (Cat #sc-15404) were purchased from Santa Cruz Biotechnology (Santa Cruz). The antibodies for FOXO3 (Cat #2497), phosphor-FOXO3 (Ser25) (Cat #13129) and beta-actin (Cat #4970) were obtained from Cell Signaling Technologies. The antibody to cyclophilin A (Cat #07-313) was from Upstate Biotechnology. Blots were analyzed with a LAS-3000 imaging system (Fujifilm).

### siRNA treatment

H9c2 cells were transfected using DharmaFECT 4 (Dharmacon, cat #T-2004-02) with 100 nM ON-TARGETplus SMARTpool for FOXO3 (Cat #L-095006-02-0020) and SIRT1 (Dharmacon, cat #L-094699-02-0010). After 24 hours, cells were washed with PBS, exposed to 16h hypoxia followed by reoxygenation and treatment with syringaresinol for 9 h and harvested for mRNA analysis.

### Immunoflorescence Microscopy

H9c2 cells were fixed with 3% formaldehyde in PBS, permeabilized with 70% ethanol, and incubated with anti-FOXO3a (diluted 1:100, Cell Signaling) antisera in a microscopy buffer (2% BSA and 0.1% Triton X-100 in PBS), followed by Alexa 488-conjugated donkey anti-rabbit IgG (Molecular Probes Inc., cat #A-21206). DNA was stained with 4′,6-diamidino-2-phenylindole (DAPI, cat #D3571). The specimens were observed with a Zeiss Axioplan-2 microscope. Image acquisition and post-processing were performed with Northern Eclipse software (Empix).

### Statistical analysis

All the experiments were repeated for at least six or three times. Data were expressed as mean ± SD. Normality of data was analyzed by Shapiro–Wilk test, and comparison of results between different groups was performed by One-way (followed by Tukey's multiple comparison tests) or Student's *t*-test with SPSS 12.0 software (SPSS). Statistical significance was determined at a value of *P* < 0.05.

## SUPPLEMENTARY MATERIAL FIGURE



## Abbreviations

Bnip3: Bcl-2/adenovirus E1B 19kDa protein-interacting protein 3
Ctrl: control
CVD: cardiovascular disease
H/R: hypoxia/reoxygenation
HIF: hypoxia-inducible factor
I/R: ischemia/reperfusion
LDH: lactate dehyrogenase
mPTP: mitochondrial permeability transition pore
ROS: reactive oxygen species
SYR: syringaresinol.
