# Supplementary material for: Syringaresinol protects against hypoxia/reoxygenation-induced cardiomyocytes injury and death by destabilization of HIF-1α in a FOXO3-dependent mechanism
Source: Oncotarget. 2014 Nov 6;6(1):43–55. doi: 10.18632/oncotarget.2723 (PMC4381577; doi:10.18632/oncotarget.2723)
Supplement: Supplementary file 1 [file oncotarget-06-043-s001.pdf]

Syringaresinol protects against hypoxia/reoxygenation-induced cardiomyocytes injury and death by destabilization of HIF-1α in a FOXO3-dependent mechanism

Supplementary Material

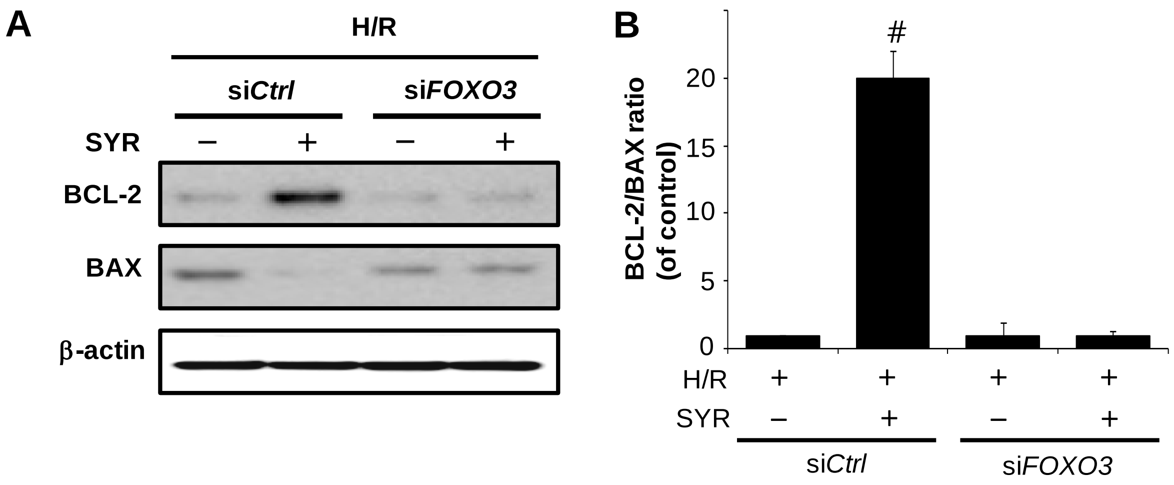

Supplementary Figure 1: FOXO3 knockdown abolishes the Syringaresinol-induced change in BCL-2/BAX ratio after H/R. H9c2 cells were transfected with control or *FOXO3* siRNA. After 24hr, cells were exposed to H/R in the presence and absence of syringaresinol. (A) Expression levels of apoptotic-related proteins, BCL-2 and BAX were examined by Western blot. Actin levels are shown as loading controls. (B) BCL-2/BAX ratios were assessed. All results are representative or means ± SD of four independent experiments. Ctrl: control; H/R: hypoxia/reoxygenation; SYR: syringaresinol. #P < 0.001 versus control group.
